# Scleral Buckle Infection with Aspergillus Flavus

**DOI:** 10.4103/0974-9233.53371

**Published:** 2008

**Authors:** Manal Bouhaimed, Hassan Al-Dhibi, Abdullah Al-Assiri

**Affiliations:** 1 From the Department of Surgery and Community Medicine, Faculty of Medicine, Kuwait University, and Vitreoretina Division, AlBaher Eye Center, Ministry of Health, Kuwait; 2 From the Department of Vitreoretina Division, King Khaled Eye Specialist Hospital, Riyadh, Kingdom of Saudi Arabia; 3 From the Department of Anterior Segment Division, King Khaled Eye Specialist Hospital, Riyadh, Kingdom of Saudi Arabia

**Keywords:** scleral buckle infection, Aspergillus, extruded buckle

## Abstract

**Purpose:**

To present a case of scleral buckle infection with Aspergillus flavus in a tertiary eye center in Saudi Arabia.

**Methods:**

A retrospective case report of a 28-year-old Saudi male who presented with a six-month history of conjunctival injection and discharge from the left eye which had undergone uncomplicated conventional retinal detachment surgery, at the King Khaled Eye Specialist Hospital in Riyadh, Saudi Arabia, in the form of cryopexy, subretinal fluid drainage and scleral buckle (grooved segmental sponge and circumferential band with sleeve) for a macula on retinal detachment four years earlier. A diagnosis of infected extruded scleral buckle was made and the buckle was removed.

**Results:**

The infected scleral buckle was removed under local anesthesia with administration of sub-conjunctival irrigation of 50 mg solution of Vancomycin, and sub-conjunctival injection of 25mg of Vancomycin. Post operative microbiological studies revealed infection with silver staining of moderate Aspergillus flavus hyphae. Visual acuity of the left eye improved from 20/200 before surgery to 20/60 in the two years follow-up visit.

**Conclusion:**

This case report indicates the importance of considering infection with multiple organisms – including fungal ones – in cases of scleral buckle infections in our population.

Although pars plana vitrectomy is increasingly used for primary repair of rhegmatogenous retinal detachment, conventional methods with cryopexy and scleral buckle are still used in 37 to 64.4% of cases.^1^

Reported rates of removal of a scleral buckle for different indications vary from 3.8 to 24.0 %.^2^ Infections and extrusion of the scleral buckle were the most common reasons for buckle removal cited in the literature.^3^ In a large series of 757 patients who undergone cryotherapy and scleral buckle for rhegmatogenous retinal detachment; removal of the implant was necessary in 10 patients (1.3%). Silicone sponge (9% of 32 patients) was more frequently removed than was silicone rubber (0.6% of 360 patients) or MIRAgel (1.3% of 386 patients).^4^

The most common organisms causing scleral buckle infection are coagulase positive and coagulase negative Staphylococci species; these account for 70 to 90% of all organisms identified,^5^ however, isolated cases with infections due to atypical mycobacterium, corynebacteria and fungi have been reported. Here we report a case of scleral buckle infection with Aspergillus flavus from a tertiary eye center in the Kingdom of Saudi Arabia.

## Case Report

A 28-year-old Saudi male from the Southern province underwent uncomplicated conventional retinal detachment surgery, at the King Khaled Eye Specialist Hospital in Riyadh, Saudi Arabia, in the form of cryopexy, subretinal fluid drainage and scleral buckle (grooved segmental # 505 sponge and circumferential # 240 band with # 270 sleeve) for a macula on retinal detachment in the left eye.

The patient had undergone renal transplant for chronic renal failure three years earlier. In addition, he had hypertension and normocytic normochromic anemia secondary to his renal condition and was otherwise stable on hemodialysis twice weekly.

Four years after the retinal detachment surgery, he presented with a six-month history of conjunctival injection and discharge from the left eye.

**Figure 1:**
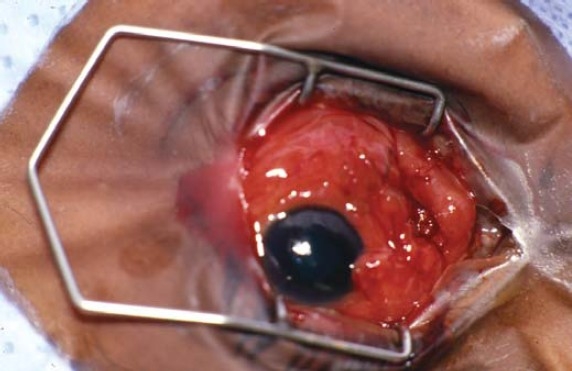
Severely hyperemic conjunctiva and an exposed buckle.

On examination, visual acuity in his left eye was 20/200 and intraocular pressure (IOP) was 14mmHg. The conjunctiva was severely hyperemic and an exposed buckle could be seen inferonasally (Fig. 1) with white discharge around it. The cornea was clear and the anterior chamber was deep and quiet. Dilated fundus examination showed a flat retina.

**Figure 2:**
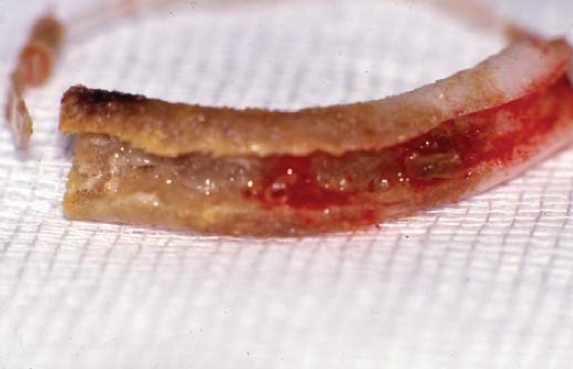
Discoloration of explanted buckle.

**Figure 3:**
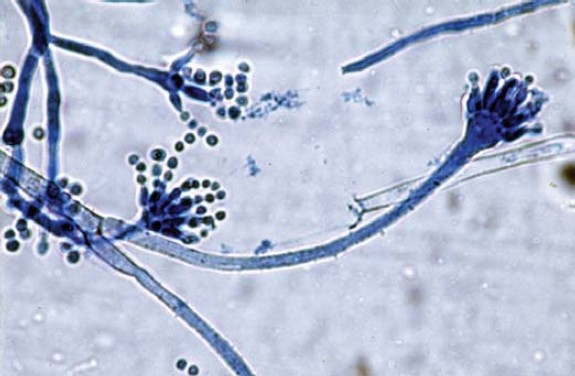
Silver staining of Aspergillus flavus hyphae.

The infected scleral buckle was removed under local anesthesia; it was intact with visible discoloration (Fig. 2). Sub-conjunctival irrigation with 50 mg solution of Vancomycin, and sub-conjunctival injection of 25mg of Vancomycin were given. Microbiological studies revealed infection with methicillin resistant Staphylococcus aureus (MRSA) and silver staining of moderate Aspergillus flavus hyphae (Fig. 3).

The patient was treated postoperatively for 1 month with topical Vancomycin 50mg/ml, Bacitracin ointment and Predforet drops. Visual acuity improved gradually to 20/60 and the conjunctival appearance returned back to normal with flat retina on fundus examination. The patient was followed up for 2 years before being discharged from the hospital.

## Discussion

Scleral buckle infection following retinal reattachment surgery is infrequent complication. In this case, however, the patient had specific risk factors which predisposed him to acquire such a rare infection with a fungal element. Firstly, the patient presented with an exposed buckle increasing his risks significantly of acquiring an infection. In a report by Smiddy et al,^6^ 45 cases of scleral buckle infection among approximately 3000 scleral buckling procedures performed at their institution between July 1, 1985 and July 1, 1991 were identified. The scleral buckle was exposed in all 45 cases.

Secondly, in determining susceptibility to Aspergillosis, the host immune defenses are of crucial importance. Although Aspergillosis may be seen in normal individuals, invasive infection is much more common in immunocompromised hosts. A variety of conditions, including hematological malignancies, administration of chemotherapy and immunosuppressive agents, and neutropenia may predispose to severe infections with Aspergillus. The history of renal failure and treatment with immunosuppressive medications in our patient is therefore very significant.

Thirdly, a report by Ismail and Abdel-Sater in 1994^7^ indicated that Aspergillus can grow under a wide range of environmental conditions in Saudi Arabia, and the temperature and humidity from January to March are optimal for the growth and multiplication of Aspergillus species such as Aspergillus fumigatus. Whether our patient lived in an area in the Kingdom with such climatic predisposition was not determined.

Other significant environmental factors such as those associated with hospital construction projects, demolition of old buildings and yard work have been implicated in dissemination of fungal spores. In 1996, Aspergillus endophthalmitis was reported postoperatively in a series of 5 patients in Saudi Arabia in a hospital during a period of old buildings construction.^8^

In our patient, there was no evidence of intraocular infection; only conjunctival involvement was seen with infection of the exposed buckling element. Finally, although the use of processed human donor pericardium patch grafting is one useful way to avoid removing exposed scleral buckles, it was excluded in this case because of the long post operative duration (4 years) reducing the risk of recurrent retinal detachment and the clinically evident infection.
